# Protocol of a systematic review on the application of wearable inertial sensors to quantify everyday life motor activity in people with mobility impairments

**DOI:** 10.1186/s13643-018-0824-4

**Published:** 2018-10-24

**Authors:** Fabian Marcel Rast, Rob Labruyère

**Affiliations:** 1 Rehabilitation Center for Children and Adolescents, University Children’s Hospital Zurich, Mühlebergstrasse 104, CH-8910 Affoltern am Albis, Switzerland; 2 Children’s Research Center, University Children’s Hospital Zurich, University of Zurich, Zurich, Switzerland; 3 Rehabilitation Engineering Laboratory, Department of Health Sciences and Technology, ETH Zurich, Zurich, Switzerland

**Keywords:** Disabled persons, Patients, Rehabilitation, Accelerometer, Gyroscope, Inertial measurement unit, Algorithms, Pattern recognition, Machine learning, Activities of daily living

## Abstract

**Background:**

People with mobility impairments may have difficulties in everyday life motor activities, and assessing these difficulties is crucial to plan rehabilitation interventions and evaluate their effectiveness. Wearable inertial sensors enable long-term monitoring of motor activities in a patient’s habitual environment and complement clinical assessments which are conducted in a standardised environment. The application of wearable sensors requires appropriate data processing algorithms to estimate clinically meaningful outcome measures, and this review will provide an overview of previously published measures, their underlying algorithms, sensor placement, and measurement properties such as validity, reproducibility, and feasibility.

**Methods:**

We will screen the literature for studies which applied inertial sensors to people with mobility impairments in free-living conditions, described the data processing algorithm reproducibly, and calculated everyday life motor activity-related outcome measures. Three databases (MEDLINE, EMBASE, and SCOPUS) will be searched with terms out of four different categories: study population, measurement tool, algorithm, and outcome measure. Abstracts and full texts will be screened independently by the two review authors, and disagreement will be solved by discussion and consensus. Data will be extracted by one of the review authors and verified by the other. It includes the type of outcome measures, the underlying data processing algorithm, the required sensor technology, the corresponding sensor placement, the measurement properties, and the target population. We expect to find a high heterogeneity of outcome measures and will therefore provide a narrative synthesis of the extracted data.

**Discussion:**

This review will facilitate the selection of an appropriate sensor setup for future applications, contain recommendations about the design of data processing algorithms as well as their evaluation procedure, and present a gap for innovative, new algorithms, and devices.

**Systematic review registration:**

International prospective register of systematic reviews (PROSPERO): CRD42017069865.

**Electronic supplementary material:**

The online version of this article (10.1186/s13643-018-0824-4) contains supplementary material, which is available to authorized users.

## Background

People with mobility impairments may have difficulties in executing activities of daily living (activity limitations), or they may experience problems in involvement in life situations (participation restrictions) [1]. Rehabilitation services aim to improve these people’s abilities or make changes to their environment [2], to achieve a high level of independence and eventually increase the quality of life. Clinical assessments to estimate patients’ abilities and their rehabilitation progress are generally conducted in a standardised environment at a single time. Thus, they do not incorporate environmental and cognitive challenges of a patient’s habitual environment [3] and might be inaccurate when the symptoms of the patient fluctuate over time [4]. Recent advances in wearable sensor technologies enable objective and long-term monitoring of motor activities in a patient’s habitual environment. They provide an opportunity to overcome the aforementioned limitations of clinical assessments and complement their outcome measures.

Accelerometers and pedometers are the most commonly used wearable devices to quantify everyday life motor activity in clinical trials and clinical practice [5, 6]. Conventional outcome measures of accelerometers are activity counts as well as intensity levels and energy expenditure estimations based on cut points of these counts [7]. These measures provide relevant information about whole body physical activity, but they are non-specific and cannot determine movement patterns and types of activities performed [8]. Pedometers recognise walking activities and count the number of steps during a day. However, they reveal reduced accuracy in people with altered gait patterns and slow walking speeds [6, 9].

In contrast, using a combination of several inertial sensors, such as accelerometers and gyroscopes, together with sophisticated data processing algorithms allows estimating the quantity and quality of everyday life motor activities [10]. Additional sensor technology such as magnetometers, barometers, wearable cameras, and heart rate monitors measure environmental factors or physiological responses to motor activities and can be combined with inertial sensors to gain further details about patients’ activities [11, 12]. Technological progress in the field of microelectromechanical systems has made these devices small-sized, cost-effective, energy-efficient, and thus applicable for continuous long-term monitoring in unsupervised, free-living conditions [10]. However, the analysis of this tremendous amount of unlabelled raw data requires appropriate data processing algorithms to determine clinically meaningful outcome measures of everyday life motor activity. Examples of such measures are a hand use laterality index [13], a ratio between active and passive wheelchair propulsion [14], and a number of daily climbed stairs [15].

The relevance of these outcome measures depends on end users’ perspectives and may be different for people with mobility impairments compared to non-disabled individuals. For example, the amount of limping, use of assistive devices, and daily activity of affected limbs are more relevant to the first population. Altered movement patterns can also be a challenge for data processing algorithms [16, 17] and thus the transferability of algorithms which were evaluated in non-disabled individuals to people with mobility impairments could be limited. Therefore, this review will focus on the application of inertial sensor technologies to quantify everyday life motor activity in people with mobility impairments. It will provide an overview of existing outcome measures and their underlying data processing algorithms. Specifically, the following research questions will be addressed:

1. Which outcome measures have been used to quantify everyday life motor activity of people with mobility impairments under free-living conditions and what are their corresponding data processing algorithms?
2. Which inertial sensor technology (accelerometer or gyroscope), possibly in combination with additional wearable sensor technology, is required to assess these measures?
3. Where need inertial sensors be placed to assess these measures and minimally restrict activities of daily living?
4. In which patient populations were these measures applied and were they evaluated in terms of validity, reproducibility, or feasibility?

## Methods/design

This protocol was registered with the International prospective register of systematic reviews (PROSPERO) in June 2017 (registration number: CRD42017069865). The development and reporting of this protocol are in accordance with the checklist of the Preferred Reporting Items for Systematic Review and Meta-Analysis Protocols (PRISMA-P) [18].

### Eligibility criteria

We will include full-text articles written in English or German if they meet all of the following eligibility criteria. There will be no restrictions on year of publication:

#### Measurement tool

The described system incorporates an accelerometer, a gyroscope, or both and can optionally include additional sensors such as a magnetometer, a barometer, a wearable camera, and a heart rate monitor. All required devices must be body worn or attached to assistive devices (e.g. wheelchair). If the system relies on data from external (e.g. a smart home environment) or implanted devices (e.g. instrumented prosthesis), the article will be excluded.

#### Algorithm

The algorithm describes the data processing of recorded raw data up to the resulting outcome measure. The algorithm must be described reproducibly in the article, or references providing this information must be cited and publicly available. In addition, the algorithm must be applicable to unlabelled data of unrestricted, unsupervised long-term measurements. If an algorithm only works with predetermined movement recordings and thus with labelled data, such as in clinical gait analysis, the corresponding article will not be included in this review.

#### Outcome measure

The output of the data processing algorithm must be a measure that quantifies an aspect of everyday life motor activity (e.g. number of reaching activities, gait symmetry, or use of assistive devices). Whole body activity counts and step counts, as well as physical activity levels and energy expenditure based on thresholds of these counts, will not be considered for this review, as they have already been well investigated [19, 20] and provide no innovation compared to the current clinical state of the art. Metrics that quantify an emergency situation (e.g. epileptic seizure or fall detection), a non-mobility-related activity (e.g. sleep or food intake), or a disease-specific motor behaviour (e.g. freezing of gait in Parkinson’s disease) will be excluded as well.

#### Study population

We will include all articles that analysed data from children, adolescents, or adults with a diagnosed orthopaedic or neurological mobility impairment (e.g. cerebral palsy, stroke, osteoarthritis) or from those who need assistive devices in their daily life activities (e.g. crutches, wheelchairs). Study populations with mental or visual impairments as well as patients suffering from cardio-respiratory conditions will not be considered, as we assume that these populations do not present an altered movement pattern in everyday life motor activities compared to healthy controls. Infants will be excluded since they pose different requirements to a monitoring device for motor activities. Exceptions are possible if an article introduces a novel algorithm with highly relevant outcome measures for people with mobility impairments, but only preliminary data with healthy subjects are available.

### Search strategy

We will conduct a systematic search of the literature in three databases: MEDLINE via the Ovid search engine including in-process and other non-indexed citations as well as EMBASE and SCOPUS via Elsevier’s search engine. A preliminary search was conducted in July 2017 and will be repeated before completion of the review article.

The selected search terms can be grouped into five categories: (1) study population, (2) measurement tool, (3) data processing algorithm, (4) free-living condition, and (5) terms which incorporate categories three and four. The first category limits the search results to articles with a clinical application. It comprises both general terms (e.g. “patient”, “disease”, “rehabilitation”) as well as specific health conditions (“spinal cord injury”, “stroke”). The second category includes the most frequently used synonyms of inertial sensors (“accelerometer”, “gyroscope”, “inertial measurement unit”). The third category restricts the search results to articles containing a description of the data processing algorithms with terms such as “algorithm”, “signal processing”, and “pattern recognition”. The search terms of the fourth category were selected to find algorithms that are applicable in free-living conditions (e.g. “everyday life”, “daily living”). The last category comprises two terms “activity classification” and “activity recognition”. An OR operator will be used to link search terms within categories, while an AND operator will be used between categories. The final search strategy combines the categories as follows: [(1) AND (2) AND (3) AND (4)] OR [(1) AND (2) AND (5)]. Search fields will be used to restrict the search to title, abstract, and keywords. If applicable, medical subject headings (MeSH) and terms of the Emtree thesaurus will be used in the corresponding search engines. The complete list of search terms and the syntax of the search strategy are provided in Additional file 1.

### Selection process

Titles and abstracts of all articles retrieved using the search strategy described above will be screened by the two review authors independently to identify articles that potentially meet the eligibility criteria. The full text of these potentially eligible studies will be retrieved and independently assessed for eligibility by the same review authors. Disagreements will be resolved by discussion and consensus. For the data management of the selection process, we will use Covidence, a Cochrane technology platform [21].

### Data extraction

Data extraction from all included articles will be conducted by one of the review authors and checked by the other. Extracted information will include outcome measures and method of the underlying data processing algorithm, type and placement of required sensor technology, study design, and evaluation of the outcome measures, as well as study population. Discrepancies will be identified and resolved through discussion and consensus. Missing data will be requested from the authors of the respective article.

### Data synthesis

The purpose of this review is to provide an overview of all published outcome measures that quantify everyday life motor activity. Most likely, they will be grouped in activity-independent (e.g. hand use laterality) and activity-dependent measures, which could be further subdivided into quantity (e.g. duration of sitting activities, number of climbed stairs) and quality measures (e.g. symmetry index of walking activities). We will conduct a narrative synthesis of the methods that were used to assess these outcomes. This will include the type and placement of required sensor technologies and a brief description of the underlying data processing algorithms. Further, we will provide an overview of how these measures were evaluated. This will cover the study population, the study design, and the type of analysis (e.g. validity, reproducibility, or feasibility). Our systematic review will provide readers with extensive information about measurement of everyday life motor activities in patient populations with wearable sensors, and the presentation of the information will be divided into several categories, like outcome measures, sensor setup and technology, diagnosis, and study type.

## Discussion

We expect to find a high heterogeneity of outcome measures to quantify everyday life motor activity and different study designs to evaluate them. Our preliminary search revealed that there would be mainly four different types of studies in our review: (1) case-control studies that assessed the discriminant validity of its outcome measures, (2) clinical validity studies that correlated their outcome measures with a standardised clinical assessment in a specific patient population, (3) studies that evaluated the activity classification accuracy of their algorithm, and (4) concurrent validity studies that investigated the error of their outcome measures by comparing the outcomes of the wearable sensor technology with a reference method. These study types reveal different test statistics and cannot be compared with each other. The comparison between studies will be further complicated since they include different study populations. All this impedes a quantitative synthesis of the study results. Therefore, the primary purpose of this systematic review will be to provide a comprehensive overview of the methods of previous studies instead of synthesising their results. Accordingly, it will grant researchers quick access to all studies that evaluated a specific outcome measure in a particular patient population.

Advances in wearable sensor technology enable long-term monitoring of everyday life motor activities in people with mobility impairments. This monitoring potentially provides important information to the rehabilitation process, as it describes the patient’s motor abilities in his/her habitual environment. Many different devices and corresponding data processing algorithms have been developed over the last decade, and this review will provide an overview of these methods with a focus on outcome measures and clinical applications. It will facilitate the selection of an appropriate sensor setup for future applications and present a gap for innovative, new algorithms and devices.

## Additional file

Additional file 1: Search strategy (PDF 362 kb)

## Abbreviations

MeSH: Medical subject headings
PRISMA-P: Preferred Reporting Items for Systematic Review and Meta-Analysis Protocols
PROSPERO: International prospective register of systematic reviews
