# Supplementary material for: Protocol of a systematic review on the application of wearable inertial sensors to quantify everyday life motor activity in people with mobility impairments
Source: Syst Rev. 2018 Oct 24;7:174. doi: 10.1186/s13643-018-0824-4 (PMC6201500; doi:10.1186/s13643-018-0824-4)
Supplement: Supplementary file 1 — Search strategy (PDF 362 kb) [file 13643_2018_824_MOESM1_ESM.pdf]

UNIVERSITY
CHILDREN'S HOSPITAL
ZURICH
Rehabilitation Centre
Affoltern am Albis

ETH zürich

RELAB
Rehabilitation Engineering Lab

Search strategy

"Systematic review on the application of wearable inertial sensors to quantify everyday life motor activity in people with mobility impairments"

Search categories and Boolean operators

1) People with mobility impairments
2) Inertial sensor technology
3) Data processing algorithms
4) Free-living conditions
5) Activity recognition or classification
6) Animal studies

{1 AND [(2 AND 3 AND 4) OR (2 AND 5)]} NOT 6

Database-specific search terms

Medline (Ovid)

1) exp disabled persons/ or exp motor skills disorders/ or exp rehabilitation/ or exp patients/ or exp "diseases (non mesh)"/ or ("handicap*" or "disab*" or "disorder*" or "rehabilitation*" or "disease*" or "patients" or "motor impairment*" or "dysfunction*" or "syndrome*" or "brain injur*" or "spinal cord injur*" or "palsy" or "paralysis" or "*paresis" or "hypertonia" or "spasticity" or "meningitis" or "myopath*" or "neuropath*" or "stroke").ti,ab,kw.

2) "actigraph*".ti,ab,kw. or exp accelerometry/ or "actimetry".ti,ab,kw. or "acceleromet*".ti,ab,kw. or "gyroscope".ti,ab,kw. or "magnetomet*".ti,ab,kw. or exp magnetometry/ or "inertial sensor*".ti,ab,kw. or "inertial measurement unit*".ti,ab,kw. or "imu".ti,ab,kw. or "motion sensor*".ti,ab,kw. or "movement sensor*".ti,ab,kw.

3) ("algorithm*" or "signal process*" or "data process*" or "pattern recogni*").ti,ab,kw. or exp computing methodologies/ or exp Pattern Recognition, Automated/

4) "daily living*".ti,ab,kw. OR "daily life".ti,ab,kw. OR "adl".ti,ab,kw. OR "everyday life".ti,ab,kw. OR "free living".ti,ab,kw. OR "outdoor*".ti,ab,kw. OR "home".ti,ab,kw. OR "hand activit*".ti,ab,kw. OR "arm activit*".ti,ab,kw. OR "walking activit*".ti,ab,kw. OR (daily ADJ3 activit*).ti,ab,kw. OR exp "Activities of Daily Living"/

5) ((classif* adj3 activit*) or (recogni* adj3 activit*)).ti,ab,kw.

6) exp animal/ not exp human/

Embase

1) 'disabled person'/exp or 'disability'/exp or 'rehabilitation'/exp or 'patient'/exp or 'diseases'/exp or ('handicap*' or 'disab*' or 'disorder*' or 'rehabilitation*' or 'disease*' or 'patients' or 'motor impairment*' or 'dysfunction*' or 'syndrome*' or 'brain injur*' or 'spinal cord injur*' or 'palsy' or 'paralysis' or 'paresis' or 'hypertonia' or 'spasticity' or 'meningitis' or 'myopath*' or 'neuropath*' or 'stroke'):ti,ab,de

2) 'actigraph*':ti,ab,de OR 'actimetry'/exp OR 'actimetry':ti,ab,de OR 'acceleromet*':ti,ab,de OR 'accelerometry'/exp OR 'accelerometer'/exp OR 'gyroscope':ti,ab,de OR 'magnetomet*':ti,ab,de OR 'magnetometry'/exp OR 'inertial sensor*':ti,ab,de OR 'inertial measurement unit*':ti,ab,de OR 'imu':ti,ab,de OR 'motion sensor*':ti,ab,de OR 'movement sensor*':ti,ab,de

3) 'algorithm*':ti,ab,de OR 'signal process*':ti,ab,de OR 'data process*':ti,ab,de OR 'pattern recogni*':ti,ab,de OR 'information processing'/exp OR 'signal processing'/exp

4) 'daily living*':ti,ab,de OR 'daily life':ti,ab,de OR 'adl':ti,ab,de OR 'everyday life':ti,ab,de OR 'free living':ti,ab,de OR 'outdoor*':ti,ab,de OR 'home':ti,ab,de OR 'hand activit*':ti,ab,de OR 'arm activit*':ti,ab,de OR 'walking activit*':ti,ab,de OR (daily NEAR/3 activit*):ti,ab,de OR 'daily life activity'/exp

5) (classif* NEAR/3 activit*):ti,ab,de OR (recogni* NEAR/3 activit*):ti,ab,de

6) 'animal'/exp NOT 'human'/exp

Scopus

1) TITLE-ABS-KEY ("handicap*" OR "disab*" OR "disorder*" OR "rehabilitation*" OR "disease*" OR "patients" OR "motor impairment*" OR "dysfunction*" OR "syndrome*" OR "brain injur*" OR "spinal cord injur*" OR "palsy" OR "paralysis" OR "*paresis" OR "hypertonia" OR "spasticity" OR "meningitis" OR "myopath*" OR "neuropath*" OR "stroke")

2) TITLE-ABS-KEY("actigraph*" OR "actimetry" OR "acceleromet*" OR "gyroscope" OR "magnetomet*" OR "inertial sensor*" OR "imu" OR "inertial measurement unit*" OR "motion sensor*" OR "movement sensor*")

3) TITLE-ABS-KEY("algorithm*" OR "signal process*" OR "data process*" OR "pattern recogni*")

4) TITLE-ABS-KEY ("daily living*" OR "daily life" OR "adl" OR "everyday life" OR "free living" OR "outdoor*" OR "home" OR "hand activit*" OR "arm activit*" OR "walking activit*" OR (daily W/2 activit*))

5) TITLE-ABS-KEY ((classif* W/2 activit*) OR (recogni* W/2 activit*))

6) INDEXTERMS (animal AND NOT human)
